# NanoLuc-based microneutralization assay for high-throughput detection of neutralizing antibodies against rift valley fever virus

**DOI:** 10.1016/j.mex.2026.103948

**Published:** 2026-05-07

**Authors:** Cigdem Alkan, Alexander N. Freiberg, Tetsuro Ikegami

**Affiliations:** a Department of Pathology, The University of Texas Medical Branch at Galveston, 301 University Blvd., Galveston, TX 77555, USA; b The Sealy Institute for Vaccine Sciences, The University of Texas Medical Branch at Galveston, 301 University Blvd., Galveston, TX 77555, USA; c The Center for Biodefense and Emerging Infectious Diseases, The University of Texas Medical Branch at Galveston, 301 University Blvd., Galveston, TX 77555, USA

**Keywords:** Rift valley fever virus, Microneutralization assay, NanoLuc, MP-12 strain, Neutralization antibody, Assay development

## Abstract

Plaque reduction neutralization tests (PRNTs) are the gold standard for measuring neutralizing antibodies but are labor-intensive, low-throughput, and subject to variability from manual plaque counting. We developed a NanoLuc-based microneutralization (mNT) assay using rMP12-NanoLuc that quantifies luminescence in culture supernatants as a surrogate for infection. The assay runs in 96-well plates without overlay, fixation, or staining, using 35 focus-forming unit (FFU) per well and a 40 hour readout with back-titration to ensure accuracy. mNT titers from vaccinated animal sera showed strong correlation with conventional PRNTs. This approach enables rapid, reproducible, high-throughput detection of RVFV neutralizing antibodies, with the main limitations being substrate cost and the requirement for a luminometer.

• The assay enables high-throughput, objective neutralization testing in 96-well plates.
• Optimized conditions yield mNT titers that strongly correlate with conventional PRNTs.
• Limitations include NanoLuc substrate cost and the need for a luminometer.

## Graphical abstract

**Figure:**
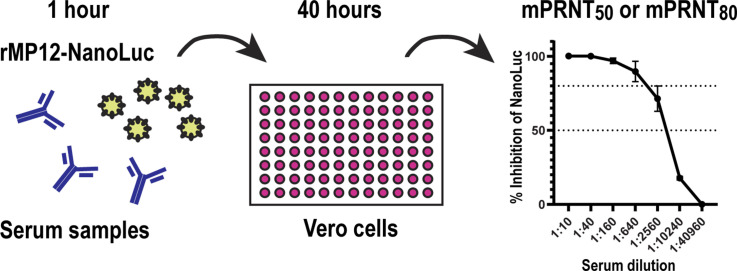

## Background

Rift Valley fever (RVF) is a mosquito-borne zoonotic virus endemic to parts of Africa and the Arabian Peninsula [1]. The causative agent, Rift Valley fever virus (RVFV), is a negative-strand RNA virus of the genus *Phlebovirus* within the family *Phenuiviridae*, with a tripartite genome consisting of Large (L), Medium (M), and Small (S) segments [2]. Infected livestock, including sheep, cattle, and goats, experience high rates of abortion, fetal loss, and mortality, while humans may develop symptoms ranging from mild febrile illness to severe complications such as retinitis, encephalitis, or hemorrhagic fever [3]. Since neutralizing antibodies play a major protective role against severe disease caused by RVFV infection, their presence is considered an important parameter for predicting the protective status of vaccinated humans and animals [4,5]. In addition, the presence of neutralizing antibodies provides highly specific evidence of past RVFV infection, with minimal risk of cross-reactivity with other viruses. The Plaque Reduction Neutralization Test (PRNT) is a standard assay for detecting neutralizing antibodies induced by RVFV infection or vaccination. The traditional PRNT assay involves: (1) pre-incubation of serially diluted sera with RVFV at 37 °C for 1 hour; (2) infection of Vero or Vero E6 cells with the serum–virus mixture at 37 °C for 1 hour; (3) removal of the inoculum and addition of an overlay; (4) incubation at 37 °C for 72 h; (5) removal of the overlay followed by cell fixation and staining with crystal violet; and (6) counting plaques to calculate the percent inhibition relative to control wells without serum. In addition, the readout is prone to error because manual plaque counting is subjective and depends on the operator’s experience, which can affect result accuracy. Recently, a recombinant RVFV strain (RVFV_56/74) expressing NanoLuc, in which NanoLuc is self-cleaved from the N protein or NSs protein via porcine teschovirus (PTV) 2A sequences, was reported by others [6,7]. The rRVFV_56/74-Nluc is a Risk Group 3 pathogen that requires high-containment (BSL-3+) facilities. It expresses the NSs gene, induces strong cytopathic effects in infected cells, and retains virulence in animals, making it suitable for antiviral efficacy studies. Because NanoLuc is expressed in infected cells, its activity in culture supernatants can be used to detect neutralizing antibodies in microneutralization (mNT) assays, similar to assays previously developed using other reporter systems [[8], [9], [10]].

In this study, we evaluated a NanoLuc-based mNT assay using a recombinant RVFV MP-12 vaccine strain expressing the NanoLuc reporter protein in place of the NSs protein. NanoLuc is secreted from infected cells via the human IL-6 signal sequence, allowing direct measurement of reporter activity in culture supernatants using 96-well plates. This virus is classified as a Risk Group 2 pathogen, exempt from select agent regulations, and can be handled under BSL-2 conditions. This mNT assay eliminates the need for overlays and manual plaque counting, but requires optimization of viral input, incubation time, and methods for determining neutralization titers, which are addressed in this report.

### Method details

Preparation of recombinant virus:

1. Plasmid DNA
  • The plasmids used for the rescue of rMP12-NanoLuc were as follows: pProT7-vS(+)-NanoLuc (expressing full-length MP-12 S-segment RNA with NanoLuc in place of NSs), pProT7-vM(+) (expressing full-length MP-12 M-segment RNA), pProT7-vL(+) (expressing full-length MP-12 L-segment RNA), pT7-IRES-vN (expressing MP-12 N protein), pT7-IRES-vL (expressing MP-12 L protein), and pCAGGS-vG (expressing MP-12 GnGc protein). The preparation of plasmids for rescuing MP-12 has been described previously [11].
  • The NanoLuc ORF (VFTLEDF…) was fused to an upstream signal sequence derived from the human IL-6 gene (MNSFSTSAFGPVAFSLGLLLVLPAAFPAP).
2. Cells and media for virus rescue
  • Baby hamster kidney (BHK) cell–derived BHK/T7–9 cells [12] or BSR-T7/5 cells [13], constitutively expressing T7 RNA polymerase: Either BHK-T7/9 or BSR-T7/5 cells were used to rescue rMP12-NanoLuc. Cells were maintained in Minimum Essential Medium (MEM) Alpha supplemented with 10 % Fetal Bovine Serum (FBS), 100 units/ml of Penicillin, 100 µg/ml of Streptomycin, and 600 µg/ml of puromycin (for BHK/T7–9 cells) or 1 mg/ml of G418 (for BSR-T7/5). Both cell types support the rescue of rMP12-NanoLuc.
  • Vero cells (ATCC CCL-81): Vero cells were used to re-amplify the rescued rMP12-NanoLuc virus and to perform virus titration. Cells were maintained in Dulbecco’s Modified Eagle Medium with high glucose, supplemented with 10 % fetal bovine serum (FBS), 100 U/ml penicillin, and 100 µg/ml streptomycin (hereafter referred to as DMEM).
  • Cells were maintained at 37 °C in 5 % CO_2_.
3. The rescue of rMP12-NanoLuc virus: Virus was rescued using a previously described reverse genetics system [11]. Briefly, Baby Hamster Kidney (BHK) cells stably expressing T7 RNA polymerase: e.g., BHK-T7/9 cells [12] or BSR-T7/5 cells[13],.at 80–90 % confluency in 60-mm dishes were transfected with 2.2 μg each of pProT7-vS(+)-NanoLuc, pProT7-vM(+), and pProT7-vL(+), as well as pT7-IRES-vN (2.2 μg), pT7-IRES-vL (1.1 μg), and pCAGGS-vG (1.1 μg). The DNA was mixed with transfection reagent (TransIT-293 or TransIT-LT1, Mirus) at a ratio of 1:2 to 1:3 (DNA:reagent). Culture supernatant was replaced at 24 h post transfection and was harvested at 5 days post transfection. Subsequently, the rescued rMP12-NanoLuc virus was re-amplified in Vero cells at a multiplicity of infection (MOI) of 0.01–0.001 for 3–4 days.
4. Titration of rMP12-NanoLuc virus: Vero cells in 6-well plates at 90–95 % confluency were infected with 400 µl per well of 10-fold serial dilutions of rMP12-NanoLuc virus at 37 °C for 1 hour. After removing the inoculum, the cells were overlaid with a medium containing 0.3 % tragacanth gum, 1× MEM, 5 % FBS, 5 % triphosphate broth, 100 U/ml penicillin, and 100 µg/ml streptomycin. At 48 h post-infection (hpi), the overlays were removed, and the cells were fixed with methanol and acetone (1:1) at room temperature for 15 min. After removal of the fixative, the cells were immunostained with an anti-RVFV antibody (e.g., mouse ascites from the World Reference Center for Emerging Viruses and Arboviruses at UTMB, or anti-RVFV N rabbit polyclonal antibody) [14], followed by Alexa Fluor 488–conjugated secondary antibody. Infectious virus titers (focus-forming units [FFU] per ml) were calculated using the formula: plaque number × dilution × 1000 / 400, where 400 represents the inoculum volume (µl).
  • The overlay was prepared by mixing equal volumes of 0.6 % tragacanth gum and 2× MEM containing 10 % FBS, 10 % triphosphate broth, 100 U/ml penicillin, and 100 µg/ml streptomycin.
5. Dilution of rMP12-NanoLuc for mNT assay: The original virus stock titer was 5.5 × 10^6^ FFU/ml. To allow further optimization of virus input in mNT assay, the stock was diluted 76-fold (2 ml virus plus 150 ml DMEM), yielding a final concentration of approximately 3.6 × 10^4^ FFU/ml (36 FFU/µl). The titer of diluted virus was back-titrated and determined as 88 FFU/µl.

Microneutralization (mNT) assay:

1. In 96-well plates, sera were subjected to 4-fold serial dilution in DMEM. Aliquots (10 µl) of each dilution were mixed with 2.5 µl of rMP12-NanoLuc, after which the plates were sealed and incubated at 37 °C for 1 hour. In addition to serum samples, at least three no-serum wells (DMEM + virus) were included as controls.
  • **Serial dilution:** Serum (3 µl) was mixed with 21 µl DMEM, and 10 µl of this mixture was transferred into 30 µl DMEM to generate 4-fold serial dilutions starting at 1:8. Each diluted serum sample (10 µl) was then mixed with 2.5 µl of rMP12-NanoLuc virus, yielding final serum dilutions of 1:10, 1:40, and subsequent 4-fold dilutions in the reaction mixture. In addition, 10 µl of DMEM was mixed with 2.5 µl of rMP12-NanoLuc virus as a control in at least three separate wells. Due to the small handling volumes, the virus was first spotted at the left bottom of each well in 96-well plates. Sera were then added to the virus and gently mixed at the left bottom of the well to ensure thorough mixing. Although this procedure is designed for use with flat-bottom plates, V-bottom plates may also be suitable.
  • **Adjustment of input virus in a defined reaction volume:** The diluted rMP12-NanoLuc virus stock had a concentration of 88 FFU/µl. To prepare the virus working solution, 1 ml of virus stock was mixed with 1.5 ml of DMEM (final dilution, 1:2.5), yielding a concentration of 35.2 FFU/µl. Subsequently, 2.5 µl of this virus mixture was added to each well, resulting in 88 FFU per reaction.
  • **Use of no serum wells as controls:** In this mNT assay, NanoLuc values from wells containing virus without serum were used to establish the maximum values for each input virus. Serum samples from non-vaccinated individuals served as negative controls, but they were not used to set the assay threshold in order to minimize inter-assay variation caused by nonspecific inhibitory effects of normal sera.
2. Vero cells (80–95 % confluency at infection) were seeded in 96-well tissue culture plates with 95 µl of DMEM per well. The 5 µl serum–rMP12-NanoLuc mixture was then added to each well, resulting in a final volume of 100 µl per well.
  • **Final input virus used for infection:** The input virus corresponded to 5 µl of a 12.5 µl mixture (88 FFU), resulting in a final dose of 35.2 FFU per well.
  • **Preparation of Vero cell monolayer:** Vero cells should be seeded at least 1 hour prior to infection. Cell seeding density per well should be optimized by each operator depending on the interval prior to infection.
3. At 40 hpi, culture supernatants were collected into a separate 96-well plate and stored at −80 °C.
  • **Handling of culture supernatants:** Culture supernatants contain infectious rMP12-NanoLuc and must be handled in a BSL-2 laboratory using appropriate containment measures.
  • **Sample storage at –80** °**C:** Culture medium containing secreted NanoLuc can be stored at –80 °C prior to the addition of Nano-Glo substrate. To minimize loss of luciferase activity, freeze–thaw cycles should be avoided, and NanoLuc activity should be measured promptly.
4. For validation of the optimized viral input and measurement timing, viral inocula of 18 and 88 FFU per well, along with NanoLuc measurements at 20 and 64 hpi, were also evaluated.
5. NanoLuc activity in culture supernatants was measured using the Nano-Glo Luciferase Assay System (Promega) according to the manufacturer’s instructions. For each well of a 96-well white half-area microplate (Revvity), 10 µl of water, 2.5 µl of culture supernatant, and 12.5 µl of reconstituted Nano-Glo® Luciferase reagent were added. Blank wells containing 12.5 µl of water and 12.5 µl of reconstituted Nano-Glo Luciferase reagent should be included in at least three separate wells. Plates were sealed, and luminescence was measured at UTMB using a Cytation 7 plate reader (Agilent) for 96-well plates or a GloMax 20/20 luminometer (Promega) for individual tubes.
  • Reconstitution of Nano-Glo Luciferase Assay Substrate: The Nano-Glo Luciferase Assay Substrate was mixed with 50 vol of Nano-Glo Luciferase Assay Buffer. The reconstituted reagent was used immediately, and NanoLuc activity was measured within 1 hour of preparation.
6. The relative light units (RLU) from NanoLuc activity were converted into percent inhibition. Percent inhibition was calculated using the formula:
  • % inhibition = ((RLU_virus only_ − RLU_sample_) ÷ (RLU_virus only_ − RLU_blank_)) × 100

ᵒ RLU_sample_ is the luminescence from wells containing serum and virus
ᵒ RLU_virus only_ is from wells containing virus only
ᵒ RLU_blank_ is from wells without virus or serum.

7. The serum dilutions corresponding to 50 % or 80 % inhibition (mNT_50_ and mNT_80_, respectively) were calculated by interpolation using the following formula:
  • mNT_50_ = log_10_(lower dilution) + (50 − lower % inhibition) ÷ (higher % inhibition − lower % inhibition) × (log_10_(higher dilution) − log_10_(lower dilution))
  • mNT_80_ = log_10_(lower dilution) + (80 − lower % inhibition) ÷ (higher % inhibition − lower % inhibition) × (log_10_(higher dilution) − log_10_(lower dilution))

ᵒ The percent inhibition used in the formula was the mean value of triplicate measurements.

Standard Plaque Reduction Neutralization Test (PRNT) for the comparison with mNT assay:

A standard PRNT was performed to compare neutralizing antibody titers with those obtained from the mNT assay. The standard PRNT uses the parental rMP-12 virus, which does not express NanoLuc but produces clear plaques on Vero cell monolayers. The protocol was as follows:

1. In 96-well plates, sera were subjected to 4-fold serial dilution in DMEM. Aliquots (20 µl) of each dilution were mixed with 5 µl of rMP-12, after which the plates were sealed and incubated at 37 °C for 1 hour. In addition to serum samples, at least three no-serum wells (DMEM + virus) were included as controls.
  • **Serial dilution:** Serum (6 µl) was mixed with 42 µl DMEM, and 20 µl of this mixture was transferred into 60 µl DMEM to generate 4-fold serial dilutions starting at 1:8. Each diluted serum sample (20 µl) was then mixed with 5 µl of rMP-12 virus, yielding final serum dilutions of 1:10, 1:40, and subsequent 4-fold dilutions in the reaction mixture.
  • **Adjustment of input virus in a defined reaction volume:** The diluted rMP-12 virus stock had a concentration of 50–60 plaque-forming units (PFU) per 5 µL.
2. The serum–rMP-12 mixture (25 µL) was diluted with 150 µL of DMEM. A 150 µL of this mixture was then added to Vero cells (80–95 % confluency) in 24-well plates and incubated at 37 °C for 1 hour.
3. After removal of the inoculum, cells were overlaid with a medium (500 µL per well) containing 0.3 % tragacanth gum, 1× MEM, 5 % FBS, 5 % triphosphate broth, streptomycin, and penicillin, and then incubated at 37 °C for 72 h.
  • The overlay was prepared by mixing equal volumes of 0.6 % tragacanth gum and 2× MEM containing 10 % FBS, 10 % triphosphate broth, 100 U/ml penicillin, and 100 µg/ml streptomycin.
4. The overlay was removed, and the cells were fixed and stained at room temperature for 25 min with 25 % formalin and 5 % ethanol containing crystal violet.
5. The plates were gently washed with running tap water and allowed to dry. The number of plaques per well was then counted manually.
6. The number of plaques was converted into percent inhibition. Percent inhibition was calculated using the following formula based on the observed plaque number (PN):
  • % inhibition = ((PN_virus only_ − PN_sample_) ÷ (PN_virus only_ − PN_blank_)) × 100
7. The serum dilutions corresponding to 50 % or 80 % inhibition (PRNT_50_ and PRNT_80_, respectively) were calculated by interpolation using the following formula:
  • PRNT_50_ = log_10_(lower dilution) + (50 − lower % inhibition) ÷ (higher % inhibition − lower % inhibition) × (log_10_(higher dilution) − log_10_(lower dilution))
  • PRNT_80_ = log_10_(lower dilution) + (80 − lower % inhibition) ÷ (higher % inhibition − lower % inhibition) × (log_10_(higher dilution) − log_10_(lower dilution))

Test sera:

1. All sheep and cattle sera were obtained from Dr. John Morrill, as described in his published studies [15,16].
  • Serum #21 was obtained from an ewe subcutaneously vaccinated with 1 × 10^5^ PFU of arMP12-ΔNSm21/384, collected at 69 days post-vaccination (dpv).
  • Serum #25 and #26 were obtained from ewes subcutaneously vaccinated with 1 × 10^5^ PFU of MP-12, collected at 69 dpv.
  • Serum #38 was obtained from an ewe subcutaneously vaccinated with 1 × 10^2^ PFU of arMP12-ΔNSm21/384, collected at 67 dpv.
  • Serum #95 was obtained from a cow subcutaneously vaccinated with 1 × 10^3^ PFU of arMP12-ΔNSm21/384, collected at 84 dpv.
  • Serum #107 was obtained from a cow subcutaneously vaccinated with 1 × 10^5^ PFU of arMP12-ΔNSm21/384, collected at 56 dpv.
  • Serum R233 and R239 were obtained from an unvaccinated cow and an unvaccinated sheep, respectively.
2. Seven C57BL/6 mouse sera with low neutralizing antibody titers (PRNT_80_ = 1:12, 1:12, 1:24, 1:15, 1:14, 1:18, and 1:29, PRNT_50_ = 1:71, 1:129, 1:178, 1:58, 1:33, 1:133, and 1:317, respectively) were selected from a separate vaccine study; these samples were collected 21 dpv with a single dose RVF mRNA vaccine candidate (unpublished).

Software:

1. Microsoft Excel: This software was used to calculate percent inhibition of NanoLuc RLU or plaque numbers, and to estimate interpolated values between two dilutions.
2. GraphPad Prism 10: This software was used to generate graphs and assess correlations between PRNT and mNT titers.

### Method validation

#### Evaluation of NanoLuc activities released from cells infected with rMP12-NanoLuc

**Fig. 1:**
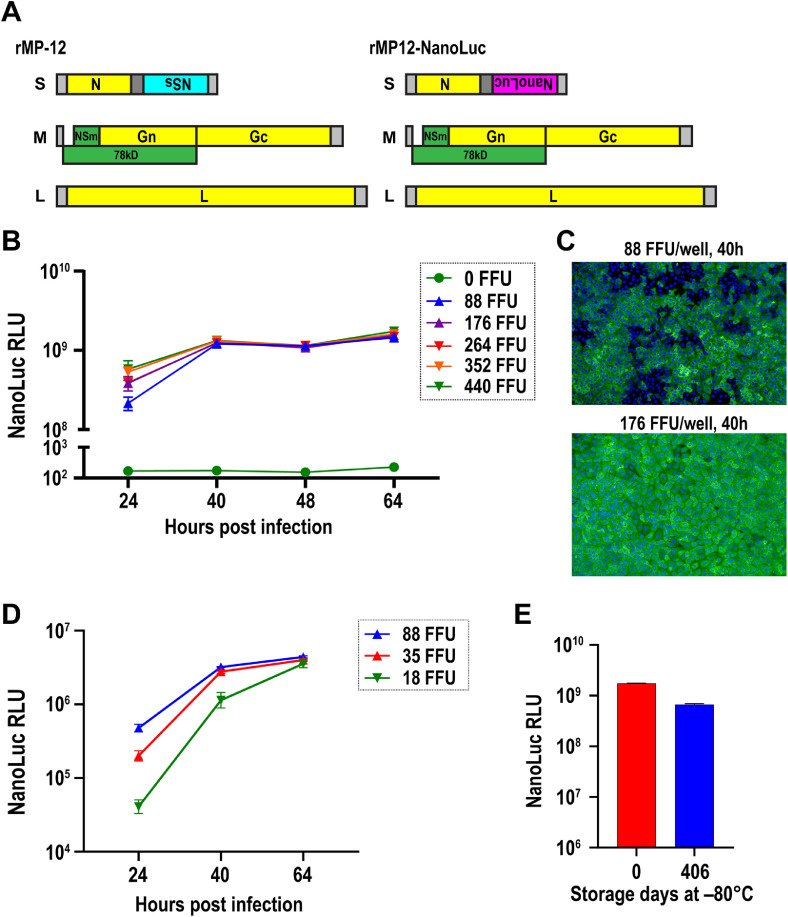
Virus dose and time point optimization for NanoLuc signal saturation. (A) Schematic representation of the S-, M-, and l-segments of parental recombinant MP-12 (rMP-12) and rMP12-NanoLuc. (B) Relative light units (RLU) in culture supernatants of Vero cells infected with 0 to 440 FFU per well of rMP12-NanoLuc at the indicated time points. (C) Immunofluorescence detection of virus-infected Vero cells at the indicated virus inputs, 40 hpi in a 96-well plate. (D) RFU in culture supernatants of Vero cells infected with 18, 35, and 88 FFU per well of rMP12-NanoLuc at the indicated time points. (E) The graph shows the mean NanoLuc activity in culture supernatants from a 440 FFU virus input collected at 64 hpi, measured before and after 406 days of storage at –80 °C. NanoLuc was measured using a GloMax 20/20 Luminometer with 10 µL of culture supernatant (B, E) or a Cytation 7 plate reader with 2.5 µL (C).

To establish a NanoLuc enzyme-based assay for quantifying neutralizing antibody titers against RVFV, recombinant MP-12 virus, in which the NSs gene is replaced with the NanoLuc luciferase reporter (rMP12-NanoLuc), was rescued from cloned cDNA using reverse genetics (Fig. 1A). Vero cells cultured in 96-well plates were infected with 88, 176, 264, 352, or 440 FFU per well of rMP12-NanoLuc to evaluate the virus dose and time points required for saturation of NanoLuc signals. Culture supernatants were collected at 24, 40, 48, and 64 hpi, and Nano-Glo reagents were added for the measurement of NanoLuc RLUs. The RLUs in the supernatants plateaued between 40 and 64 hpi, reaching approximately 10⁹ arbitrary units (Fig. 1B). At 24 hpi, NanoLuc activity was detected in a dose-dependent manner, with wells containing 88 FFU showing the lowest signal. To confirm infection, 96-well plates were fixed at 40 hpi and stained with anti-RVFV N antibody for indirect immunofluorescence assay (IFA). Wells infected with 88 FFU contained a mixture of infected and uninfected cells, whereas most cells were infected at 176 FFU. (Fig. 1C).

We next evaluated lower viral inputs (18, 35, and 88 FFU/well) for optimization of the mNT assay in 96-well plates (Fig. 1D). Consistent with the results shown in Figs. 1B and 1C, the 88 FFU condition approached saturation at 40 hpi. NanoLuc activity with 35 FFU reached 42 %, 86 %, and 90 % of the 88 FFU signal at 20, 40, and 64 hpi, respectively, indicating that the 35 FFU/40 hpi condition operates near the upper limit of the assay’s dynamic range. To evaluate whether the assay could detect proportional reductions in virus infection, the viral input was reduced from 35 FFU to 18 FFU (51 % of the 35 FFU input). This reduction in viral input resulted in corresponding decreases in NanoLuc activity to 20 %, 41 %, and 89 % of the 35 FFU signal at 20, 40, and 64 hpi, respectively. These results indicate that the assay can detect reductions in virus infection at 40 hpi under the 35 FFU assay condition.

Because the mNT assay quantifies reductions in NanoLuc activity relative to the maximum signal detected in the absence of neutralizing antibody, the 35 FFU condition was selected to maintain comparability with conventional PRNT assays, which typically use 40–60 PFU of input virus per well. Although the 35 FFU/40 hpi condition operates near the upper limit of the assay’s dynamic range, proportional reductions in NanoLuc activity were still detected following reduction of the viral input from 35 FFU to 18 FFU.

We also evaluated the NanoLuc activity in culture supernatants (*n* = 3) from a 440 FFU virus input collected at 64 hpi (Fig. 1B) after 406 days of storage at –80 °C. As shown in Fig. 1E, the mean NanoLuc activity (RLU) after storage decreased from 1.7 × 10^9^ to 6.7 × 10^8^ (∼38.5 % of the original value), indicating that, despite substantial signal loss, NanoLuc activity remained readily detectable under these conditions.

#### Evaluation of the requirement for virus–serum preincubation in the mNT assay

**Fig. 2:**
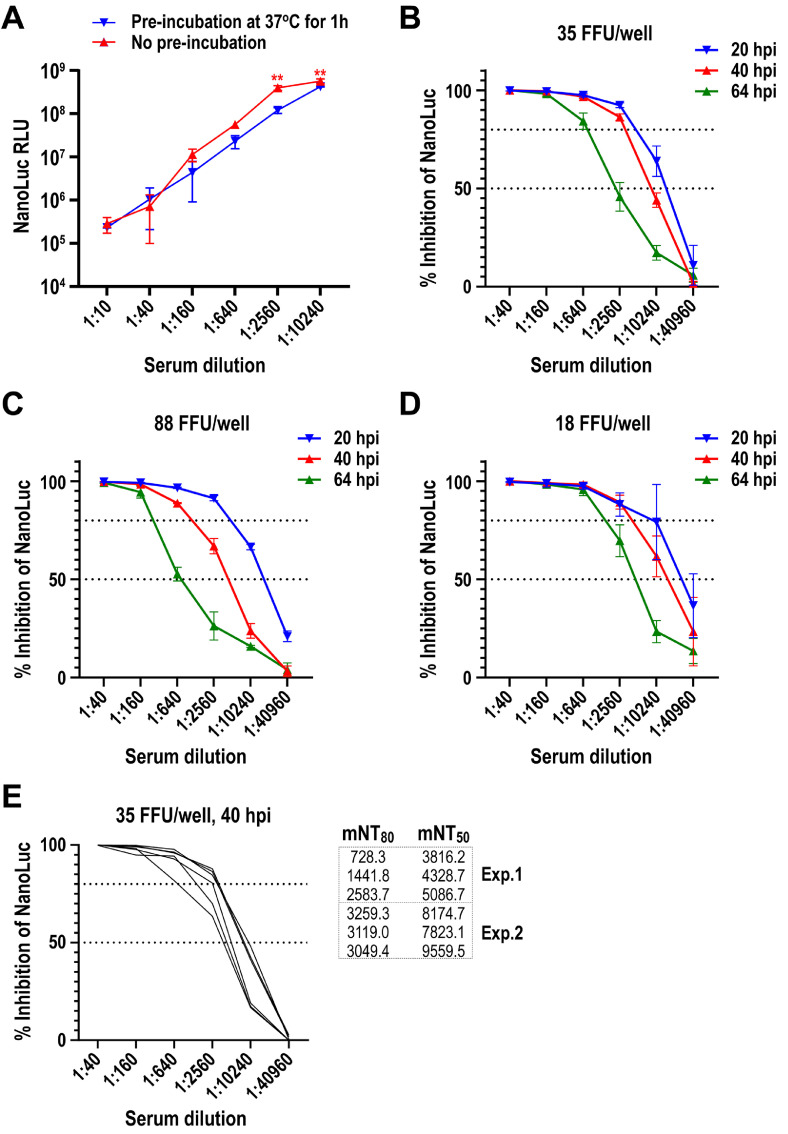
Evaluation of virus–serum preincubation, virus concentration, sample collection timing, and variability in NanoLuc values. (A) The rMP12-NanoLuc virus and diluted serum samples (in triplicate) were either (i) added directly to the culture medium without pre-incubation or (ii) pre-incubated together at 37 °C for 1 h prior to addition. NanoLuc RLU signals were measured in culture supernatants at 40 hpi. (B–D) Serially diluted serum samples from a vaccinated animal (#26), tested in triplicate, were pre-incubated with 45, 88, or 220 FFU of rMP12-NanoLuc. The mixtures were then used to infect Vero cells, with inocula containing 18, 35, or 88 FFU per well, respectively. NanoLuc RLUs were measured at 20, 40, or 64 hpi. Percent inhibition of NanoLuc RLUs (mean ± SD) was plotted for each serum sample. (E) Serially diluted serum from animal #26, tested in triplicate, was pre-incubated with 88 FFU of rMP12-NanoLuc and then used to infect Vero cells, with inocula containing 35 FFU per well. NanoLuc RLUs were measured at 40 hpi. The experiment was repeated, and individual reactions are shown in the graph. Corresponding mNT₈₀ and mNT₅₀ values for each reaction are also presented.

To optimize the mNT assay, we next evaluated whether the 1-hour virus-serum incubation at 37 °C prior to infection was necessary. In a 96-well plate without a preincubation step, 10 µl of diluted serum and 2.5 µl of rMP12-NanoLuc were added directly to Vero cells in 87.5 µl of DMEM. In a 96-well plate with a preincubation step, 10 µl of diluted serum and 2.5 µl of rMP12-NanoLuc (220 FFU/reaction) were preincubated at 37 °C for 1 hour, after which 10 µl of the mixture (176 FFU/well) was added to Vero cells in 90 µl of DMEM. As shown in Fig. 2A, NanoLuc values increased with serum dilution, regardless of whether the sera and virus were pre-incubated. At a 1:2560 dilution, NanoLuc values without pre-incubation were 3.3-fold higher than with pre-incubation, suggesting reduced virus neutralization efficiency at this dilution. Thus, we chose to perform the 1-hour virus-serum incubation at 37 °C prior to infection in subsequent assays.

#### Evaluation of viral doses and optimal timing for nanoluc measurement

Based on the Fig. 1 result, we selected 18, 35, and 88 FFU/well for mNT assay evaluation to cover a range from sub-saturating to near-saturating NanoLuc signals. Next, viral doses for incubation with serum dilutions were evaluated. During the 1-hour virus–serum incubation at 37 °C, 45, 88, or 220 FFU of rMP12-NanoLuc were used in a 12.5 µL reaction. Subsequently, 5 µL of each mixture was inoculated into Vero cells, corresponding to 18, 35, or 88 FFU per well (Fig. 2B–D). NanoLuc values were measured at 20, 40, and 64 hpi. For serum #26, the mNT_80_ and mNT_50_ titers were as follows:

• 88 FFU: 1:4813 and 1:16,917 (20 hpi), 1:1116 and 1:4410 (40 hpi), 1:258 and 1:735 (64 hpi)
• 35 FFU: 1:4672 and 1:14,727 (20 hpi), 1:3147 and 1:8417 (40 hpi), 1:746 and 1:2197 (64 hpi)
• 18 FFU: 1:8690 and 1:26,457 (20 hpi), 1:4080 and 1:15,640 (40 hpi), 1:1480 and 1:4615 (64 hpi)

Due to the absence of an overlay, unneutralized free viruses might continue spreading to remaining uninfected cells over time, leading to increased NanoLuc values and reduced NanoLuc % inhibition. Meanwhile, the input virus dose also affected the resulting mNT titers. At 20 hpi, mNT_80_ and mNT_50_ were similar for 35 and 88 FFU but were higher with 18 FFU. At 40 and 64 hpi, mNT_80_ and mNT_50_ titers were inversely related to the virus input, with higher titers observed at lower virus doses and lower titers at higher doses.

While the results indicated that mNT titers are influenced by virus input and the timing of NanoLuc measurement, the following mNT protocol was adopted for subsequent studies: in a 96-well plate, 10 µL of diluted serum was preincubated with 2.5 µL of rMP12-NanoLuc (88 FFU per reaction) at 37 °C for 1 hour. After preincubation, 5 µL of the mixture was added to Vero cells in 95 µL of DMEM, resulting in 35 FFU per well. NanoLuc values were then measured at 40 hpi. Fig. 2E shows six dilution series (one experiment with triplicates) of percent inhibition of NanoLuc activity. The ranges of mNT₈₀ and mNT₅₀ values (minimum to maximum) were 4.5- and 2.5-fold, respectively. In contrast, the differences in mean mNT₈₀ and mNT₅₀ values between Experiments 1 and 2 were 1.3- and 1.9-fold, respectively, indicating that triplicate measurements reduce technical variability.

#### Evaluation of mNT titers in comparison with PRNT titers

**Fig. 3:**
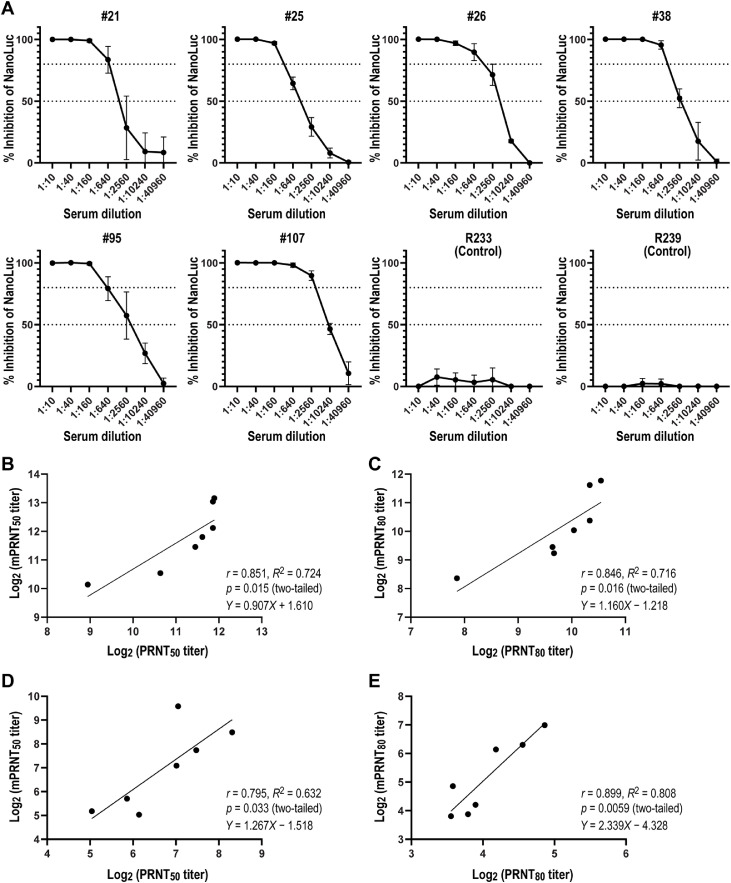
Assessment of NanoLuc-based microneutralization (mNT) titers of sera from vaccinated animals and controls. (A) Percent inhibition of NanoLuc RLUs (mean ± SD) was plotted for each serum sample. Serially diluted sera from vaccinated sheep (#21, #25, #26, and #38) and vaccinated cattle (#95 and #107), as well as unvaccinated cattle (R233) and sheep (R239), tested in triplicate, were pre-incubated with rMP12-NanoLuc and then used to infect Vero cells (35 FFU per well). NanoLuc RLUs were measured at 40 hpi. (B–E) Log₂-transformed PRNT_50_ (X-axis) versus mNT_50_ (Y-axis), and PRNT_80_ (X-axis) versus mNT_80_ (Y-axis), were plotted to assess correlations. Correlations between mNT_50_ and PRNT_50_ (B), and between mNT_80_ and PRNT_80_ (C), were evaluated for six sera from sheep and cattle (including repeated measurements for #26). Correlations for seven mouse sera are shown in (D) for mNT_50_ versus PRNT_50_ and in (E) for mNT_80_ versus PRNT_80_. Pearson correlation coefficients (r) were calculated to assess linear associations between the transformed values.

Next, six sera (#21, #25, #26, #38, #95, and #107) obtained from previous vaccination experiments [15,16], along with two control sera (R233 and R239), were tested in triplicate in the mNT assay using input virus 35 FFU per well with sample collected at 40 hpi. As shown in Fig. 3A, the six vaccinated sera exhibited strong inhibition of NanoLuc RLUs at lower serum dilutions, with inhibition gradually decreasing at higher dilutions. In contrast, the control sera showed no detectable inhibition of NanoLuc RLUs. The calculated mNT_80_ titers for sera #21, #25, #26, #38, #95, and #107 were 1:699, 1:329, 1:1332, 1:1052, 1:603, and 1:3498, respectively, while the corresponding mNT_50_ titers were 1:1488, 1:1128, 1:4446, 1:2811, 1:3574, and 1:9169, respectively. In comparison, the standard PRNT_80_ titers were 1:799, 1:232, 1:1292, 1:1052, 1:814, and 1:1492, and the PRNT_50_ titers were 1:1592, 1:494, 1:3724, 1:2800, 1:3139, and 1:3804, respectively. It was noted that the mNT_50_ and mNT_80_ titers for serum #26 were lower than those shown in Fig. 2B Therefore, these values were also included in the correlation analysis. The correlation between mNT (Y-axis) and standard PRNT (X-axis) is shown in Fig. 3B for PRNT_50_ and Fig. 3C for PRNT_80._ Log₂-transformed PRNT_80_ and mNT_80_ titers exhibited a strong positive correlation (Pearson *r* = 0.846, 95 % CI 0.256–0.977, *R*^2^ = 0.716, *p* = 0.016, two-tailed, *Y* = 1.160*X* – 0.218), indicating good agreement between the two assays. For 50 % inhibition, the correlation was similarly high (*r* = 0.851, 95 % CI 0.272–0.978, *R*² = 0.724, *p* = 0.015, *Y* = 0.907*X* + 1.610), showing a slightly lower slope but maintaining a strong linear relationship between PRNT_50_ and mNT_50_ titers.

To evaluate the correlation between mNT and PRNT at low neutralizing antibody levels, seven mouse sera were tested (Fig. 3D, E). The corresponding mNT_80_ titers were 1:14, 1:29, 1:79, 1:19, 1:15, 1:71, and 1:127, while the mNT_50_ titers were 1:33, 1:136, 1:214, 1:52, 1:36, 1:764, and 1:359, respectively. In comparison, the standard PRNT_80_ titers were 1:12, 1:12, 1:24, 1:15, 1:14, 1:18, and 1:29, and the PRNT_50_ titers were 1:71, 1:129, 1:178, 1:58, 1:33, 1:133, and 1:317, respectively. Log₂-transformed PRNT_80_ and mNT_80_ titers exhibited a strong positive correlation (Pearson *r* = 0.899, 95 % CI 0.452–0.985, *R*^2^ = 0.808, *p* = 0.006, two-tailed, *Y* = 2.339*X* – 4.328). A moderate positive correlation was observed between PRNT_50_ and mNT_50_ titers (*r* = 0.795, 95 % CI: 0.104–0.968, R² = 0.632, *p* = 0.033, two-tailed; *Y* = 1.267X – 1.518). Overall, mNT titers obtained under the condition showed positive correlations with standard PRNT titers across both high and low antibody levels, with slightly higher slopes observed at lower titers, indicating consistent assay performance.

### Limitations

Unlike conventional PRNTs, the NanoLuc based mNT measures active NanoLuc released into culture supernatants from rMP12 NanoLuc infected cells, based on the assumption that NanoLuc production reflects the number of infected cells. It should be noted that NanoLuc production approaches saturation at high levels of infection, which can compress the dynamic range of the assay. Therefore, we selected an input of 35 FFU per well and collected samples at 40 hpi. Under these conditions, proportional reductions in NanoLuc activity were still detected following reduction of the viral input from 35 FFU to 18 FFU. We also performed a back-titration of rMP12-NanoLuc after dilution of the original virus stock to maintain the assay consistency. In this regard, it is important to prepare and store diluted virus stocks so that a series of titrations within the same project can be performed using the same virus preparation, thereby minimizing assay-to assay variability.

We evaluated the requirement of preincubation of virus and serum samples. Although omission of this step would simplify the mNT assay workflow, NanoLuc activity was higher in diluted serum conditions without preincubation. Because the preincubation step determines the effective serum dilution in the virus mixture, omission of this step alters the final serum concentration; for example, mixture volumes are 100 µl without preincubation and 12.5 µl with preincubation. Therefore, we included a preincubation step when establishing the NanoLuc mNT. Using this protocol, mNT titers for six sera from previously vaccinated animals showed a strong correlation with conventional PRNT titers.

The kinetics of viral inhibition by neutralizing sera can vary depending on virus input, incubation time, and factors such as cell condition. Therefore, inclusion of both technical and biological replicates is important for detailed comparisons of mNT titers among samples, even when virus input and incubation time are standardized.

Although neutralizing antibody titers measured by mNT showed a strong correlation with PRNT, absolute values may differ between assays. Accordingly, the extent to which mNT titers correlate with protective efficacy, compared with PRNT titers, will require further validation in animal models.

## Related research article

None.

## Ethics statements

All experiments involving rMP12-NanoLuc were conducted in BSL-2 laboratory with the approval of the Institutional Biosafety Committee at UTMB, as specified in the Notification of Use (#2021017).

## CRediT authorship contribution statement

Cigdem Alkan: Writing – review & editing, Investigation, Validation, Formal analysis, Data curation. Alexander N. Freiberg: Project administration, Funding acquisition. Tetsuro Ikegami: Writing – original draft, review & editing, Methodology, Investigation, Validation, Formal analysis, Data curation, Conceptualization, Supervision, Project administration, Funding acquisition.

## Declaration of competing interest

The authors declare that they have no known competing financial interests or personal relationships that could have appeared to influence the work reported in this paper.

## Data Availability

Data will be made available on request.
